# Ovariectomy Interferes with Proteomes of Brown Adipose Tissue in Rats

**DOI:** 10.7150/ijms.66996

**Published:** 2022-03-06

**Authors:** Tzu-Jung Chou, Chia-Wen Lu, Chen-Chung Liao, Chien-Hsieh Chiang, Chi-Chang Huang, Kuo-Chin Huang

**Affiliations:** 1 Department of Family Medicine, National Taiwan University Hospital, Taipei, Taiwan.; 2 Graduate Institute of Clinical Medicine, National Taiwan University College of Medicine, Taipei, Taiwan.; 3 Department of Family Medicine, National Taiwan University College of Medicine, Taipei, Taiwan.; 4 Metabolomics-Proteomics Research Center, National Yang Ming Chiao Tung University, Taipei, Taiwan.; 5 Graduate Institute of Pharmacology, National Taiwan University College of Medicine, Taipei, Taiwan.; 6 Graduate Institute of Sports Science, National Taiwan Sport University, Taoyuan, Taiwan.; 7 Department of Family Medicine, National Taiwan University Hospital Hsin-Chu Branch, Hsinchu, Taiwan.

**Keywords:** brown adipose tissue, ingenuity pathways analysis, menopause, obesity, proteome

## Abstract

Postmenopausal women exhibit a higher prevalence of obesity due to decreased energy expenditure and increased food intake compared to their premenopausal counterparts. Brown adipose tissue (BAT) plays a key role in energy homeostasis, thus providing us with appealing therapeutic targets in obesity. However, how BAT proteomes are altered in response to low levels of estrogen remains unclear. To better understand the underlying mechanisms between the postmenopausal state and BAT proteomic changes, our study aimed to investigate the effect of ovariectomy on the BAT proteome. In this study, eight-week-old female Sprague Dawley rats were randomly allocated into bilateral ovariectomy (Ovx) and sham operation (Sham) groups. Mass spectrometry was used for proteomics assay and Ingenuity Pathway Analysis was applied to examine the differentially regulated proteins. Of the 1,412 identified proteins, 18 proteins were significantly upregulated, whereas 36 proteins were significantly downregulated in the Ovx group as compared to the Sham group. Our findings demonstrate that the proteins involved in BAT morphology, the browning of white adipose tissue, and metabolic substrates for thermogenesis were regulated by ovariectomy. The dysregulation of proteins by ovariectomy might be related to the disruption of BAT function in the postmenopausal status. Understanding how BAT proteomes are altered in response to ovariectomy may illuminate novel therapeutic strategies for the management of postmenopausal weight gain in the future.

## Introduction

Postmenopausal women tend to exhibit a higher prevalence of metabolic syndrome and obesity compared to their premenopausal counterparts 1-3. Previous studies have shown that estrogen plays a crucial role in regulating energy balance, insulin signaling, and inflammation 4, 5. Low levels of estrogen, occurring due to menopause, ovarian insufficiency, or ovariectomy (Ovx), are associated with decreased energy expenditure and increased food intake, likely leading to weight gain and obesity in the long term 6, 7. However, long-term use of hormone replacement therapy may implicate risks of breast and endometrial cancer or cardiovascular diseases in a subset of postmenopausal women 8, 9; thus, pursuing alternative solutions is needed for menopause-related obesity.

Brown adipose tissue (BAT) has become an appealing therapeutic target for obesity recently due to its distinct features of increased energy expenditure and dissipating nutrients as heat 10, 11. The abundant expression of uncoupling protein 1 (UCP1) in BAT mitochondria can generate heat by uncoupling the proton motive force from adenosine triphosphate production 12. The process is so-called non-shivering thermogenesis and can thereby increase energy expenditure 12, 13. Besides BAT, beige adipocytes also possess thermogenic capacity and can be inducible from white adipocytes, known as “browning” 14. Studies have shown that these thermogenic fat cells express estrogen receptors, supporting the notion that estrogen may play a key role in the metabolic activity of brown and beige adipocytes, and it increases UCP1 expression 15-17. In rodents, females generally have higher BAT activity than males 18. In humans, women exhibit a higher prevalence of BAT than men, but the sex difference declines with age, suggesting a possible role for the mid-life loss of estrogen in women 15. The maintenance of active BAT after the menopause has the potential to reduce the development of adiposity. However, how BAT proteomes are altered in response to low levels of estrogen remains to be determined.

Proteomic analysis is a comprehensive protein study that provides information on changes in protein expression along with their interacting networks and identifies biomarkers related to pathogenic processes 19. Proteomic techniques have demonstrated their usefulness in investigating the biological function of BAT 20, 21. To our knowledge, no comparative study on the proteomic changes of BAT in ovariectomized rats has been conducted. This study aims to investigate the effect of ovariectomy as a postmenopausal status on proteomic changes of BAT. We aim to identify molecular changes that may provide a basis for future qualitative bioinformatics analysis of mammalian BAT and shed light on therapy development for postmenopausal-related obesity.

## Materials and Methods

### Animals and experiment design

Eight-week-old female Sprague Dawley rats were obtained from BioLASCO (A Charles River Licensee Corp., Yi-Lan, Taiwan). The animals were maintained at 12 h:12 h light-dark cycles, based on previous studies that show that diurnal and circadian rhythms are related to tissue function and gene expression 22, 23. All animals were provided with a standard AIN-93 purified diet as reported 24 with some modification, including 405.7 g corn starch, 155 g dextrin, 140 g casein, 100 g sugar, 100 g corn oil, 50 g methyl cellulose, 35 g mineral mixture, 10 g vitamin mixture, 2.5 g choline bitrate, and 1.8 g L-Cystine/kg. The rats were raised at room temperature (23 ± 2°C), which was below their thermoneutral zone (28-32°C) 25, with humidity control (55% ± 10%), and offered distilled water ad libitum. The rats were allowed an acclimatization period of one week before the study was started. The animal experiment was approved by the Institutional Animal Care and Use Committee (IACUC) ethics committee under the protocol IACUC-LAC-99-251.

The rats were randomly divided into 2 groups: (1) Ovx group (n = 8), and (2) Sham control group (n = 8). The sample size of 8 rats in each group was validated by post hoc power analysis with α = 0.05 and power greater than 0.9. The effect size was calculated by Cohen's d. After acclimation for one week, anesthesia was performed by intraperitoneal injection of Zoletil/Xylazine (30 mg/kg Zoletil with 10 mg/kg Xylazine). The bilateral ovariectomy was conducted according to a standard CAF procedure. These Ovx rats only produce estrogen from extragonadal sites and were used extensively as a model of surgically induced menopause 26-28. The Sham rats were subjected to the same general surgical procedure as that of the Ovx rats, except for the removal of bilateral ovaries. Since rats may have stress-like responses to the operation, and stress can induce browning and activation of BAT 29, 30, our study used the sham operation as the control group to avoid any adverse effects of surgery on BAT. All animals underwent the operation on the same date. Bodyweight and food intake were documented in the beginning of the study and four months after ovariectomy. As described in our previous report 26-28, the success of the ovariectomy was confirmed by assessing the plasma estradiol level. The plasma estradiol level was analyzed using a radioimmunoassay kit (Diagnostic Products Corporation, Los Angeles, CA, USA). Fourth months after ovariectomy, the plasma estradiol level was significantly decreased, by 90%, in the Ovx group in comparison to the Sham group (2.14±2.46 pg/mL vs. 21.07±9.3, *p* < 0.0001).

### Brown adipose tissue collection

Animals were anesthetized with Zoletil/Xylazine and euthanized four months after ovariectomy or sham procedure. All animals were sacrificed on the same date after 12 h fasting. The interscapular BAT was carefully harvested, processed with ice-cold normal saline, then blotted dry and stored at -80 °C for further use. Blood samples were collected from the abdominal aorta.

### Protein extraction and mass spectrometric data analysis

Protein preparation, extraction, SDS-PAGE, and in-gel digestion of the BAT samples were the same as reported in our previous study 26. Each rat BAT sample (50 mg) was placed in a 1 mL sample tube containing ceramic beads and homogenized in cold buffer. Each lane of the gel was cut into ten equal pieces after G-250 staining. Each piece was de-stained in a mixed 50% acetonitrile and 25 mM NH_4_HCO_3_ (1:1, v/v) solution. After vacuum drying, gel pieces were rehydrated with 25 mM NH_4_HCO_3_ containing 1% β-mercaptoethanol and were alkylated in the buffer containing 5% 4-vinylpyridine in 25 mM NH_4_HCO_3_ and 50% acetonitrile (1:1, v/v) for 20 min. Trypsin (Promega, Mannheim, Germany) was added to digest the proteins overnight in gels in 25 mM NH_4_HCO_3_ (1%, w/v) at 37 °C. Afterward, tryptic peptides were extracted from the gel piece with 25 mM NH_4_HCO_3_ for 10 minutes, dried, and kept at -20 °C for further analysis.

Tryptic peptides dissolved in 10 μL formic acid (0.1%, v/v) were analyzed by liquid chromatography-mass spectrometry (NanoAcquity UP LC system, Waters, Manchester, UK) linked to a linear ion trap (Orbitrap; Thermo Scientific, San Jose, CA). The mobile phases consisted of solvent A (0.1% formic acid in water) and solvent B (0.1% formic acid in acetonitrile). Peptides loaded were initially desalted in a C18 PepMap column (180 μm inner diameter, 20 mm length, 5 μm beads, 100 Å pore size) and chromatographed in a C18 tip column (13.5 cm length, 75 μm inner diameter, and 5 μm beads; YMC-Gel) with 5-35% linear solvent B gradient for 90 minutes, and then 35-95% solvent B gradient for10 minutes at a flow rate of 0.5 μl/min.

The electrospray voltage applied for eluted peptides was 2 kV. Mass spectrometry (MS) data were recorded in data-dependent acquisition (isolation width: 1.5 Da), in which the *m/z* scan range was 200 to 1500 for a full scan at high resolution (> 30,000 full widths at half maximum) was scanned by MS/MS for the six most intensely charged ions (2+ and 3+). Fragmented ions of each selected precursor were generated by collision-induced dissociation with helium gas with a 35% collision energy.

The resulting MS/MS information was processed using the Xcalibur software package (version 2.0.7 SR1, Thermo-Finnigan Inc., San Jose, CA). The data were interpreted using PEAKS software (v. 8.5, Bioinformatics Solutions Inc., Waterloo, Ontario, Canada) and searched for the best-matched peptides in our protein database containing more than 530,000 entries. The false discovery rate (FDR) was set to 1% for protein identifications. All the proteins identified were annotated with UniPort ID with the following parameters: 20 ppm peptide mass tolerance and 0.8 Da fragment mass tolerance; precursor mass search type, monoisotopic; enzyme, trypsin; max missed cleavage, 2; nonspecific cleavage, 0; fixed modification; S-pyridylethylation; variable modification, methionine oxidation; and variable PTMs per peptide, 2. The results were adjusted to ≤ 1% FDR for peptide spectrum matches, -10 log P > 20, unique peptides ≥ 1, and de novo ALC score ≥ 80%. For quantitation, we normalized the MS spectral counts to the sum of the spectral counts of a sample.

### Ingenuity pathway analysis

Ingenuity Pathway Analysis (IPA) software (QIAGEN Inc., https://www.qiagenbioinformatics.com/products/ingenuitypathway-analysis) was used to investigate the functions of the BAT proteome. Analysis match, activity plot, and pattern search were performed to validate the processing of IPA. Accession numbers and expression fold change of the differentially expressed proteins were loaded into the IPA software to perform biological functions and pathway analysis. The significance (*p*-value of overlap) was calculated by the Fisher's exact test.

### Statistical analysis

All data are expressed as the mean ± standard deviation (SD) and analyzed by the t test. A *p-*value < 0.05 was considered statistically significant.

## Results

### Effects of ovariectomy on bodyweight, food intake, and calorie intake

Upon starting the experiment, there was no significant difference in initial bodyweight between the Ovx group (246±13 g) and the Sham group (240 ±14 g) at baseline. Four months after ovariectomy, the final body weight of the Ovx group (425±13 g) was greater than that of the Sham group (344±38 g) by 1.24-fold (*p*<0.05). The parametrial fat pad (PFP) and retroperitoneal fat pad (RFP) are white adipose tissues in the body. We only found that PFP mass was significantly greater for the Ovx group (17.86±3.27 g), by 1.63-fold (*p*<0.05), in comparison to that of the Sham group (10.97±2.83 g). The RFP weight had no significant difference between the Ovx group (5.70±2.73 g) and the Sham group (4.13±2.01 g). The daily food intake and calorie intake showed no significant difference between the Ovx group (12.11±1.10 g/rat/day, 38.1±7.3 kcal/rat/day) and the Sham group (10.64±2.04 g/rat/day, 43.4±3.9 kcal/rat/day). However, the daily food intake relative to body weight gain was significantly lower in the Ovx group in comparison to that of the Sham group (68.00±6.48 vs. 104.25±15.66 g/kg/day, *p* < 0.0001), which was suggestive of lower energy expenditure among the Ovx group.

### Proteomic analysis

**Figure 1:**
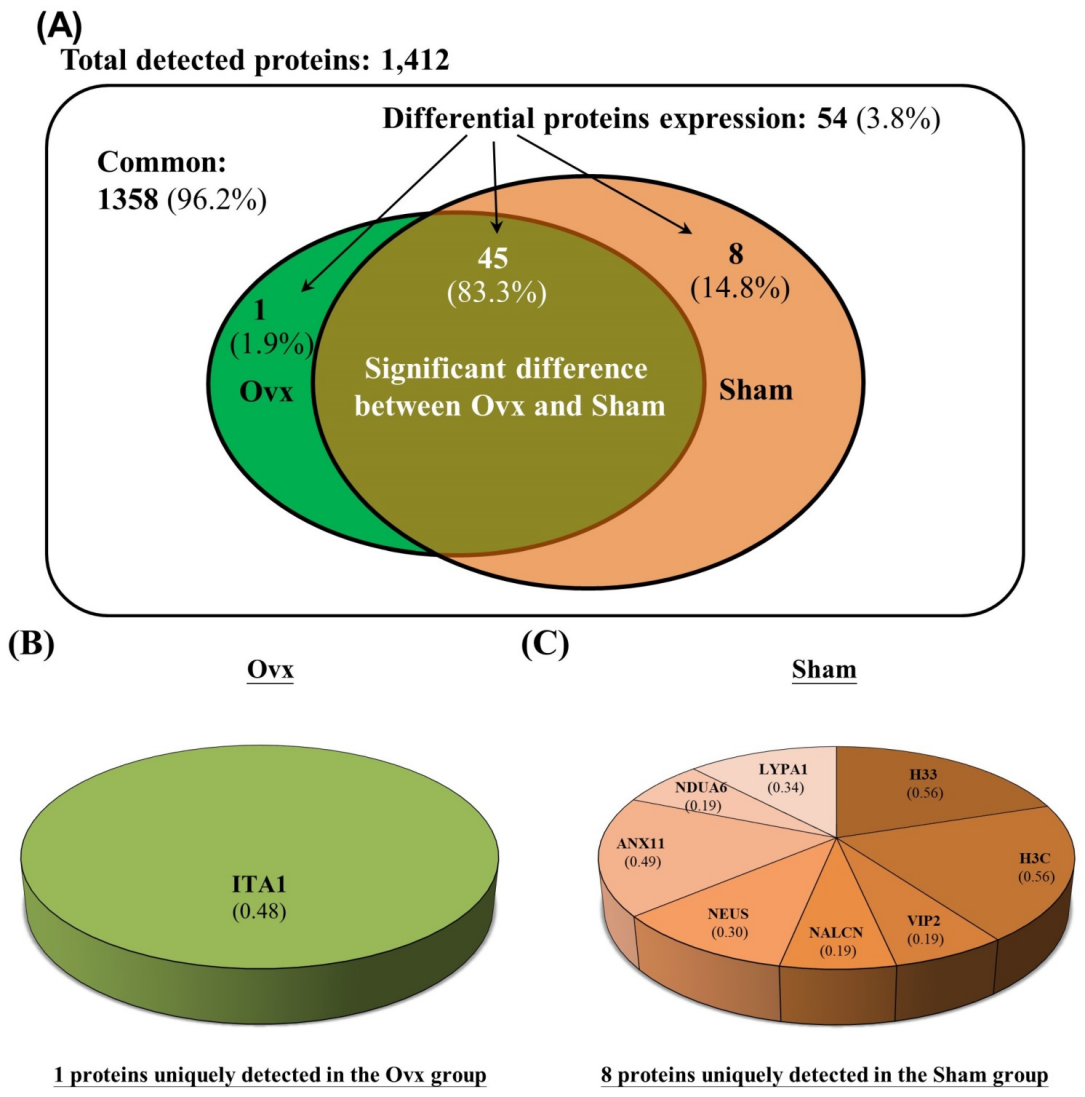
Significantly regulated proteins between the Ovx and Sham groups. (A) Venn diagram of the common, overlapping, or uniquely detected proteins; (B) The only protein detected in the Ovx group; (C) Distribution for the 8 proteins uniquely detected in the Sham group.

**Figure 2:**
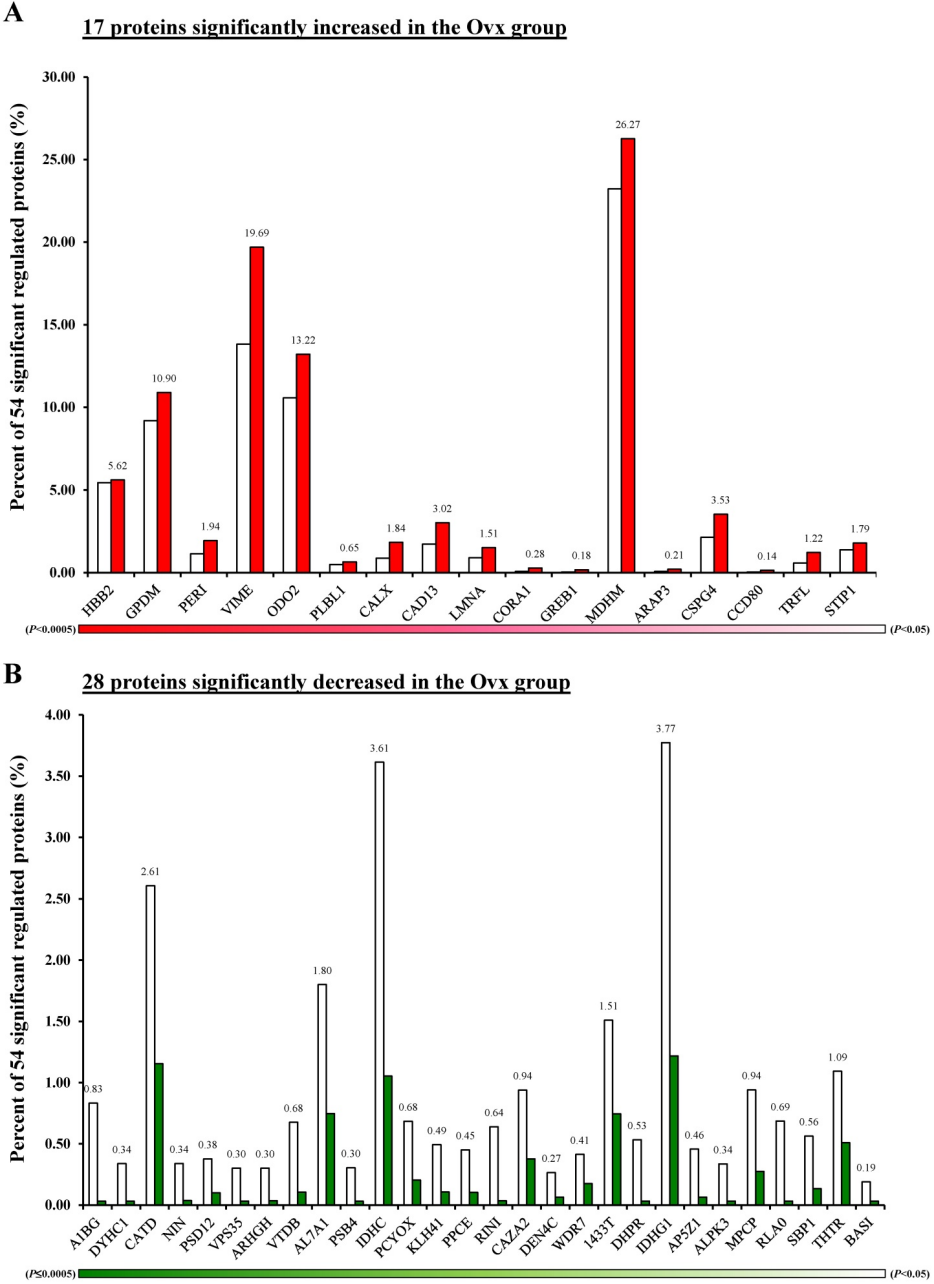
Differentially expressed proteins; (A) Percentage distribution for the 17 proteins with significantly increased in the Ovx group compared to the Sham group; (B) Percentage distribution for the 28 proteins with significantly decreased in the Ovx group compared to the Sham group.

The Venn diagram in Figure 1 shows the common, overlapping, or uniquely detected proteins in the Ovx and Sham groups. There were 96.2% (1,358/1,412) common and 3.8% (54/1,412) differentially regulated proteins. When the 1358 common proteins were deducted and the rest of 54 differentially regulated proteins were normalized to 100%, the Ovx group 1.9% (1/54) unique proteins and the Sham group showed 14.8% (8/54) unique proteins, with 83.3% (45/54) overlapping regulated proteins between the two groups (Figure 1A). The only protein expressed in the Ovx group was Integrin alpha-1 (ITA1) (Figure 1B). The eight proteins that were only expressed in the Sham group were as follows: acyl-protein thioesterase 1 (LYPA1), annexin A11 (ANX11), histone H3.3 (H33), histone H3.3C (H3C), NADH dehydrogenase [ubiquinone] 1 alpha subcomplex subunit 6 (NDUA6), inositol hexakisphosphate and diphosphoinositol-pentakisphosphate kinase 2 (VIP2), sodium leak channel non-selective protein (NALCN), and neuroserpin (NEUS) (Figure 1C). In addition, among the 45 overlapping proteins, 17 increased and 28 decreased in the Ovx group compared to the Sham group (Figure 2). A complete list of all 54 proteins showing differential expression is provided in **Table 1**.

**Figure 3:**
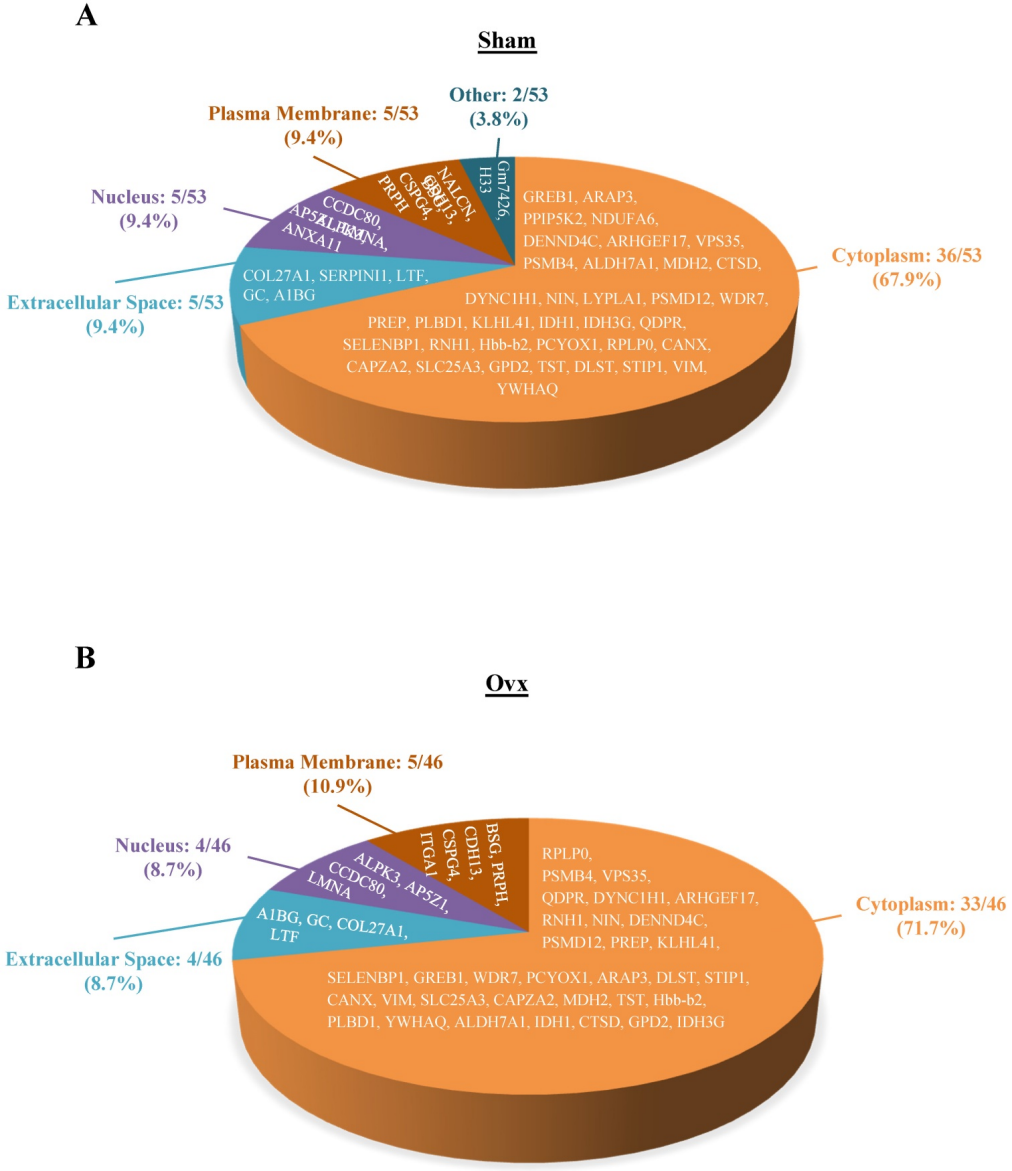
Pie charts represents the cellular locations of the differentially expressed proteins in the Ovx and Sham groups. (A) Distribution of the 53 differential proteins of the Sham group; (B) Distribution of the 46 differential proteins of the Ovx group.

The 46 and 53 differentially expressed proteins for the Ovx and Sham groups, respectively, were mostly in the cytoplasm (33/46, Ovx group; 36/53, Sham group), plasma membrane (5/46, Ovx group; 5/53, Sham group), nucleus (4/46, Ovx group; 5/53, Sham group), and extracellular space (4/46, Ovx group; 5/53, Sham group) (Figure 3).

### Ingenuity pathways analysis

**Figure 4:**
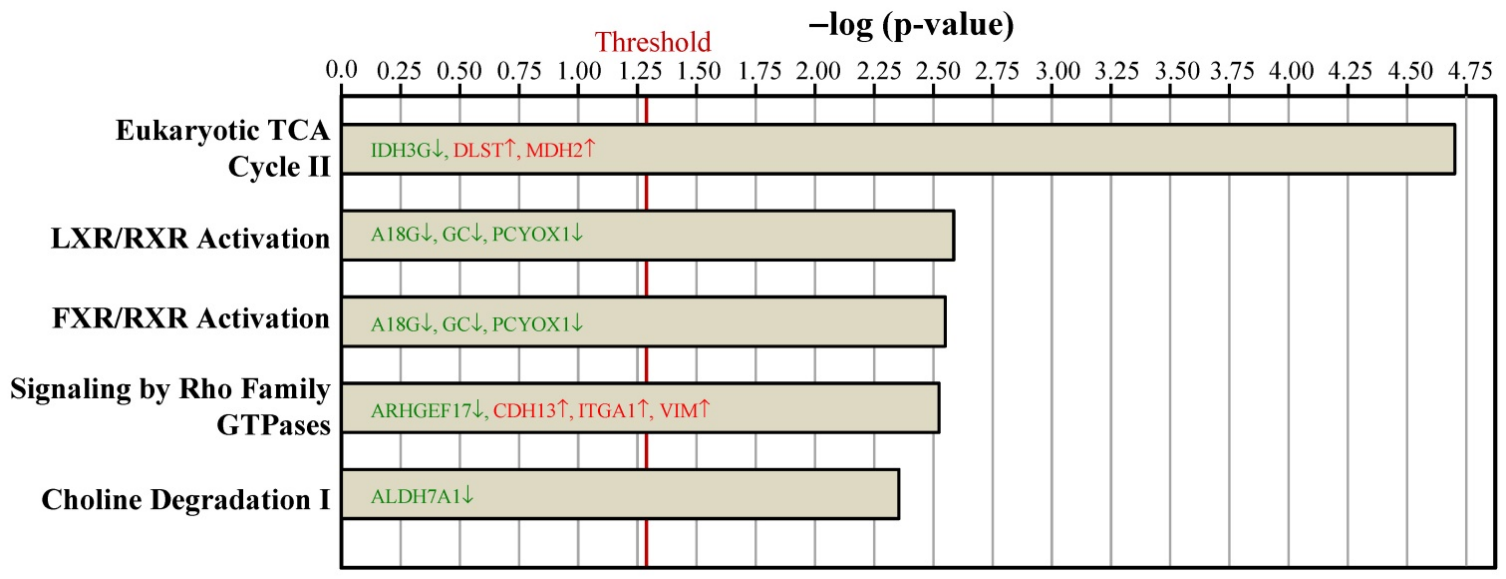
Ingenuity pathways analyses compared the top 5 canonical pathways between the Ovx and Sham groups based on peer-reviewed literature database. The x-axis represents the negative log of the *p*-value, calculated by the right-tailed Fisher exact test. Proteins in red letters were upregulated by ovariectomy and those in green letters were downregulated. The threshold of *p* < 0.05 is displayed as the red vertical line.

**Figure 5:**
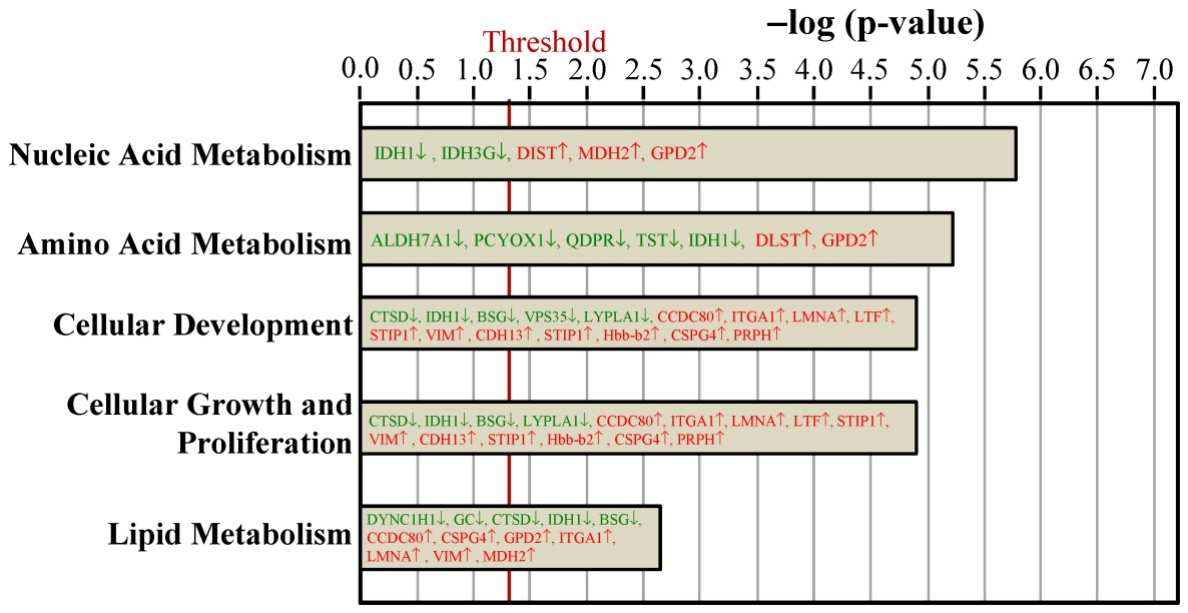
Ingenuity pathways analyses compared the biologic function proteins between the Ovx and Sham groups based on peer-reviewed literature database. The threshold of *p* < 0.05 is displayed as the red vertical line.

To examine the proteome mapping analysis of differential expressed proteins between the Ovx and Sham groups, IPA was conducted. The proteins in BAT whose expressions were changed by ovariectomy were analyzed by their canonical signaling pathways (Figure 4) and biologic functions (Figure 5). In Figure 4 and 5, the proteins in red letters were upregulated by ovariectomy, whereas those in green letters were downregulated.

The top statistically significant canonical pathways affected by the differential proteins due to ovariectomy included eukaryotic tricarboxylic acid (TCA) cycle II, liver X receptor (LXR)/retinoid X receptor (RXR) activation, farnesoid X receptor (FXR)/retinoid X receptor (RXR) activation, signaling by Rho family GTPases, and choline degradation I (Figure 4). Most of the canonical pathways were associated with energy dissipation in BAT and adipogenesis.

The key molecular and cellular functions of the ovariectomy-regulated proteins in BAT were categorized into nucleic acid metabolism, amino acid metabolism, cellular development, cellular growth and proliferation, and lipid metabolism (Figure 5). The nucleic acid metabolism influenced by differentially expressed proteins included metabolism of NADH, NADPH, and 5-hydroxymethylation of cytosine. The adipogenesis of adipose cell lines was predicted to be decreased since the VPS35 protein was downregulated in our dataset. The catabolism of amino acid and the lipolysis of non-esterified fatty acid were also affected.

## Discussion

In the current study, using the hypothesis that BAT proteins can be regulated by ovariectomy as a low level of estrogen in postmenopausal status, we performed a comparative proteomic analysis on BAT comparing Ovx and Sham rats. To gain insight into how ovariectomy interferes with BAT proteomes, IPA software was used to examine the interaction between differentially regulated proteins based on known connections in peer-reviewed literature. Although the findings are putative and preliminary, our study demonstrates that ovariectomy may impact several different proteins related to BAT metabolic pathways. The BAT proteome was significantly altered by ovariectomy, especially in proteins involving BAT morphology, browning of white adipose tissue, and metabolic substrates for thermogenesis.

Among the 54 significantly regulated proteins in our study, several proteins have been previously reported to interfere with the white adipose tissue browning pathway and disrupt the morphology of BAT. Twelve proteins were found related to the white adipose tissue browning pathway, including ARAP3, BSG, CCDC80, CTSD, GC, GPD2, IDH1, ITGA1, LMNA, LTF, LYPLA1, and VPS35. Most of these are associated with the expression of peroxisome proliferator-activated receptor-γ (PPARγ), an important transcription factor that regulates adipocyte gene expression and differentiation and induces the browning of white adipose tissue 31, 32. Estrogen receptors and PPARγ are both nuclear receptor families and have convergence of signaling crosstalk 33. Browning of white adipocytes is compromised when PPARγ signaling is disrupted due to ovariectomy 34. In addition, SBP1 and DYHC1 are involved in the differentiation of BAT 35 and were markedly reduced in the Ovx group. Decreased levels of SBP1 and DYHC1 would disrupt the morphology of BAT, causing defects in energy metabolism 36. On the other hand, the increased CSPG4 in the Ovx group leads to abnormal morphology of BAT and hinders BAT-dependent energy homeostasis 37. Our result suggests that ovariectomy hampers the browning and morphology of BAT.

A low level of estrogen reduces UCP1 expression and BAT thermogenesis in ovariectomized rats 38, 39. Two of the differentially expressed proteins, DYHC1 and LMNA, mediate sterol regulatory element binding transcription factor 1 (SREBF1) 40, 41 and CCAAT enhancer binding protein beta (C/EBP-β) 42, transcription factors that decrease the expression of mouse UCP1 mRNA 43, 44. On the other hand, transcription factors that increase expression of UCP1 such as forkhead box C2 (FOXC2) 45, E2F transcription factor 1 (E2F1) 46, and Kruppel-like factor 11 (KLF11) 47, are downregulated by multiple differentially expressed proteins in our study, including VTDB, TRFL, and VIME. In addition, several UCP1-independent thermogenic pathways have been identified in recent years, such as Ca^2+^ futile cycling, creatine futile cycling, and triglycerides-fatty acid cycling 48. Estrogen can also modulate BAT development and thermogenesis via the hypothalamic adenosine monophosphate-activated protein kinase (AMPK) signaling pathway 49. AMPK activation can stimulate Ca^2+^ cycling thermogenesis 50. We observed significantly increased lactotransferrin (TRFL), which decreases the activity of the AMPK complex 51. Moreover, LMNA and VTDB were found to alter the level of Ca^2+^ in previous murine studies 52, 53. We can therefore postulate that ovariectomy results in the disruption of UCP1 expression as well as the AMPK pathway, which leads to decreased BAT thermogenic activity in the long run.

Furthermore, estrogen may also indirectly regulate BAT activity by modulating different metabolic pathways related to energy homeostasis. Most of the differentially expressed proteins were mainly involved in nucleic acid, amino acid, and lipid metabolism. The downregulations of enzymes involved in utilizing glucose, branched-chain amino acids, and lipids for thermogenesis are of significant interest. The activation of BAT thermogenesis involves glucose uptake, branched-chain amino acid catabolism, and hydrolysis of intracellular triglycerides for the production of fatty acids, which serve as both activators and metabolic substrates for BAT mitochondria 54-56. The proteins required in the fatty acid and glucose metabolism process were found to be downregulated in the Ovx group. For instance, DYHC1 decreased in the Ovx group and would lead to decreases in lipolysis to non-esterified fatty acid 36. The catabolism of essential amino acids is affected by ODO2, AL7A1, and DHPR. In addition, proteins that act as enzymes in mitochondria were dysregulated. Specifically, IDH3G, DLST, and MDH2 have downstream effects on the metabolism of NADH in the TCA cycle 57-59. On the other hand, the uniquely upregulated protein ITA1 in the Ovx group is worth exploring. ITA1 is an important receptor for laminin and collagen in the extracellular matrix and plays a key role in cell adhesion as well as interacting with insulin receptors in adipocytes 60. The pathological accumulation of extracellular matrix proteins has metabolic consequences including insulin resistance and adipose tissue inflammation 61, 62. Furthermore, a study conducted by Malinská et al. found that the interscapular BAT in the Ovx rats exhibited decreased fatty acid oxidation, lipogenesis, and lipolysis, which might lead to visceral adiposity as well as triglyceride accumulation 63. Our results are supported by previous literature in which BAT metabolic pathways were impaired in response to ovariectomy and might have contributed to postmenopausal obesity in the long run 63, 64.

Our study has several limitations. First of all, because our study mainly focused on the BAT proteomic changes in response to ovariectomy, we did not quantify the mass of BAT nor analyze the mRNA expression. Moreover, since ovariectomized rats have been widely used as a menopausal animal model that promotes the development of obesity, glucose intolerance, insulin resistance, and dyslipidemia, we did not measure other metabolic profiles such as fasting glucose or cholesterol. The mRNA analysis is not a direct reflection of the protein content in the cell. Recent studies have indicated a poor correlation between mRNA and protein expression due to various factors such as post-transcriptional control 65, 66. An integrated transcriptomic and proteomic analysis might be warranted in the future. Secondly, the underlying mechanisms between differentially expressed proteins and BAT functions were mostly postulated from the IPA knowledge database and the known literature. Further studies building upon these preliminary findings to rigorously validate the results by thorough experiments (e.g., via histology and Western blot) are warranted. Additionally, since ovariectomy was performed in young healthy animals, the use of ovariectomized rats may not be a flawless model for natural reproductive senescence wherein aging is also an important factor. However, natural senescence in rats would increase the risks of developing cancer or other comorbidities 67, and around 60-70% of the aging rats spontaneously transition to a poly-follicular anovulatory status with low levels of progesterone and sustained levels of plasma estradiol that can last 10-100 days 68. The above-mentioned problems can thereby cause heterogeneity among our animals and further reduce the power of the experiment to detect the exclusive effect of the loss of ovarian function. Hence, we choose to use ovariectomy, a surgical model widely used in menopause that allows us to reduce possible confounding 26, 27, 69. Last but not least, although the plasma estradiol level was significantly decreased in the Ovx group, the direct causal relationship with estrogen deficiency cannot be proven due to no intervention group with estradiol to access the reversibility of these proteomic changes. Moreover, the BAT proteome might be influenced in different environments such as thermoneutrality, cold exposure, or a high-fat diet 12, 20, 70. Further studies investigating the BAT proteomic change under different temperatures or diets as well as the reversibility under estrogen replacement might be important to provide a deeper understanding of the relationship between BAT, estrogen, and environmental factors.

## Conclusions

In conclusion, we report a proteomic analysis of BAT comparing Ovx and Sham rats to investigate how the BAT proteome is altered in ovariectomized rats as a model of surgically induced menopause. Proteomic investigations into protein modulation in BAT revealed the expression of numerous proteins involved in BAT morphology, the browning of white adipose tissue, and metabolic substrates for thermogenesis was regulated by ovariectomy. The dysregulation of proteins by ovariectomy might be related to the disruption of BAT function in the postmenopausal status. Our results provide a further understanding of the effects of ovariectomy on the alterations of the BAT proteome and postulate possible links to the metabolic consequences, which may help illuminate novel therapeutic strategies for the management of postmenopausal weight gain in the future.

## Figures and Tables

**Table 1:** Differential BAT protein expression between Ovx and Sham rats ranked by *p*-values

Description	Protein-ID	Abbrev.	Sham (n=8)	Ovx (n=8)	*p* value	Fold	Pooled SD	Cohen's d	Power (1-β)
**The only protein uniquely detected in the Ovx group**									
Integrin alpha-1	Q3V3R4	ITA1	0 ± 0	3.72 ± 1.43	0.0137		0.945	3.943	0.999
**17 proteins significantly increased in the Ovx group**									
Glycerol-3-phosphate dehydrogenase, mitochondrial	Q64521	GPDM	55.12 ± 3.91	83.8 ± 8.46	0.0029	+1.52	6.164	4.653	0.999
Peripherin	P15331	PERI	6.81 ± 2.44	14.91 ± 2.66	0.0042	+2.19	2.385	3.393	0.999
Vimentin	P20152	VIME	82.86 ± 26.91	151.41 ± 26.45	0.0109	+1.83	24.959	2.747	0.999
Dihydrolipoyllysine-residue succinyltransferase component of 2-oxoglutarate dehydrogenase complex, mitochondrial	Q9D2G2	ODO2	63.37 ± 17.48	101.63 ± 11.46	0.0137	+1.60	13.825	2.767	0.999
Phospholipase B-like 1	Q8VCI0	PLBL1	2.95 ± 0.93	5.01 ± 0.35	0.0156	+1.70	0.657	3.132	0.999
Calnexin	P35564	CALX	5.25 ± 2.16	14.14 ± 4.40	0.0192	+2.69	3.244	2.740	0.999
Cadherin-13	Q9WTR5	CAD13	10.32 ± 5.76	23.26 ± 5.78	0.0193	+2.25	5.395	2.397	0.993
Prelamin-A/C	P48678	LMNA	5.38 ± 1.82	11.64 ± 3.40	0.0257	+2.16	2.550	2.455	0.995
Collagen alpha-1(XXVII) chain	Q5QNQ9	CORA1	0.45 ± 0.52	2.14 ± 0.93	0.0270	+4.80	0.705	2.405	0.993
Protein GREB1	Q3UHK3	GREB1	0.22 ± 0.45	1.35 ± 0.63	0.0309	+6.06	0.512	2.194	0.979
Malate dehydrogenase, mitochondrial	P08249	MDHM	139.24 ± 34.27	201.98 ± 28.79	0.0320	+1.45	29.604	2.119	0.970
Arf-GAP with Rho-GAP domain, ANK repeat and PH domain-containing protein 3	Q8R5G7	ARAP3	0.44 ± 0.51	1.60 ± 0.65	0.0324	+3.64	0.544	2.129	0.972
Hemoglobin subunit beta-2	P02089	HBB2	32.68 ± 6.17	43.2 ± 3.69	0.0335	+1.32	4.754	2.213	0.981
Chondroitin sulfate proteoglycan 4	Q8VHY0	CSPG4	12.83 ± 8.27	27.17 ± 5.84	0.0336	+2.12	6.699	2.141	0.973
Coiled-coil domain-containing protein 80	Q8R2G6	CCD80	0.23 ± 0.46	1.06 ± 0.07	0.0348	+4.61	0.307	2.701	0.999
Lactotransferrin	P08071	TRFL	3.47 ± 2.97	9.35 ± 3.38	0.0403	+2.70	2.973	1.980	0.946
Stress-induced-phosphoprotein 1	Q60864	STIP1	8.29 ± 3.22	13.79 ± 2.90	0.0444	+1.66	2.864	1.921	0.932
**8 proteins non-detected in the Ovx group but uniquely detected in the Sham group**									
Histone H3.3	P84244	H33	3.39 ± 0.44	0 ± 0	0.0006		0.292	11.608	0.999
Histone H3.3C	P02301	H3C	3.39 ± 0.44	0 ± 0	0.0006		0.292	11.608	0.999
Inositol hexakisphosphate and diphosphoinositol-pentakisphosphate kinase 2	Q6ZQB6	VIP2	1.12 ± 0.41	0 ± 0	0.0116		0.268	4.181	0.999
Sodium leak channel non-selective protein	Q8BXR5	NALCN	1.12 ± 0.41	0 ± 0	0.0116		0.268	4.181	0.999
Neuroserpin	O35684	NEUS	1.79 ± 0.71	0 ± 0	0.0147		0.466	3.847	0.999
Annexin A11	P97384	ANX11	2.94 ± 1.16	0 ± 0	0.0149		0.768	3.822	0.999
NADH dehydrogenase [ubiquinone] 1 alpha subcomplex subunit 6	Q9CQZ5	NDUA6	1.14 ± 0.50	0 ± 0	0.0197		0.331	3.447	0.999
Acyl-protein thioesterase 1	P97823	LYPA1	2.05 ± 1.18	0 ± 0	0.0398		0.778	2.638	0.998
**28 proteins significantly decreased in the Ovx group**									
Cytoplasmic dynein 1 heavy chain 1	Q9JHU4	DYHC1	2.03 ± 0.43	0.251 ± 0.501	0.0018	-8.09	0.437	4.070	0.999
Alpha-1B-glycoprotein	Q19LI2	A1BG	4.99 ± 1.30	0.251 ± 0.501	0.0027	-19.89	0.920	5.156	0.999
Cathepsin D	P18242	CATD	15.63 ± 2.06	8.881 ± 1.947	0.0031	-1.76	1.873	3.603	0.999
Ninein	Q61043	NIN	2.04 ± 0.48	0.287 ± 0.574	0.0037	-7.10	0.497	3.526	0.999
26S proteasome non-ATPase regulatory subunit 12	Q9D8W5	PSD12	2.26 ± 0.53	0.773 ± 0.517	0.0070	-2.92	0.490	3.033	0.999
Vacuolar protein sorting-associated protein 35	Q9EQH3	VPS35	1.81 ± 0.07	0.25 ± 0.499	0.0076	-7.23	0.333	4.673	0.999
Rho guanine nucleotide exchange factor 17	Q80U35	ARHGH	1.81 ± 0.07	0.272 ± 0.545	0.0103	-6.65	0.364	4.225	0.999
Vitamin D-binding protein	P21614	VTDB	4.05 ± 1.54	0.809 ± 0.543	0.0186	-5.01	1.077	3.012	0.999
Alpha-aminoadipic semialdehyde dehydrogenase	Q9DBF1	AL7A1	10.79 ± 2.34	5.737 ± 2.184	0.0197	-1.88	2.119	2.387	0.992
Proteasome subunit beta type-4	P99026	PSB4	1.83 ± 0.81	0.25 ± 0.499	0.0206	-7.31	0.626	2.519	0.997
Isocitrate dehydrogenase [NADP] cytoplasmic	O88844	IDHC	21.67 ± 6.94	8.109 ± 5.921	0.0257	-2.67	6.036	2.246	0.984
Prenylcysteine oxidase	Q9CQF9	PCYOX	4.10 ± 1.33	1.574 ± 1.059	0.0261	-2.61	1.122	2.252	0.984
Kelch-like protein 41	A2AUC9	KLH41	2.956 ± 0.95	0.823 ± 1.088	0.0261	-3.59	0.956	2.231	0.983
Prolyl endopeptidase	Q9QUR6	PPCE	2.70 ± 0.71	0.795 ± 1.037	0.0267	-3.40	0.831	2.298	0.988
Ribonuclease inhibitor	Q91VI7	RINI	3.83 ± 1.86	0.272 ± 0.545	0.0269	-14.09	1.284	2.773	0.999
F-actin-capping protein subunit alpha-2	P47754	CAZA2	5.63 ± 1.49	2.905 ± 0.968	0.0269	-1.94	1.175	2.317	0.989
DENN domain-containing protein 4C	A6H8H2	DEN4C	1.59 ± 0.49	0.5 ± 0.578	0.0287	-3.18	0.500	2.182	0.978
WD repeat-containing protein 7	Q920I9	WDR7	2.48 ± 0.44	1.346 ± 0.634	0.0297	-1.84	0.511	2.224	0.982
14-3-3 protein theta	P68254	1433T	9.05 ± 1.24	5.723 ± 1.87	0.0297	-1.58	1.482	2.246	0.984
Dihydropteridine reductase	Q8BVI4	DHPR	3.20 ± 1.62	0.251 ± 0.501	0.0305	-12.74	1.121	2.629	0.998
Isocitrate dehydrogenase [NAD] subunit gamma 1, mitochondrial	P70404	IDHG1	22.62 ± 7.61	9.363 ± 3.954	0.0311	-2.42	5.674	2.336	0.990
AP-5 complex subunit zeta-1	Q3U829	AP5Z1	2.74 ± 1.33	0.501 ± 1.002	0.0385	-5.47	1.099	2.037	0.957
Alpha-protein kinase 3	Q924C5	ALPK3	2.02 ± 1.10	0.25 ± 0.499	0.0409	-8.06	0.800	2.207	0.980
Phosphate carrier protein, mitochondrial	Q8VEM8	MPCP	5.64 ± 2.14	2.117 ± 1.637	0.0422	-2.66	1.780	1.979	0.946
60S acidic ribosomal protein P0	P14869	RLA0	4.12 ± 2.34	0.25 ± 0.499	0.0426	-16.46	1.583	2.443	0.994
Methanethiol oxidase	P17563	SBP1	3.38 ± 1.11	1.039 ± 1.419	0.0429	-3.25	1.190	1.966	0.943
Thiosulfate sulfurtransferase	P52196	THTR	6.55 ± 1.17	3.916 ± 1.64	0.0438	-1.67	1.333	1.977	0.945
Basigin	P18572	BASI	1.14 ± 0.50	0.251 ± 0.501	0.0457	-4.54	0.468	1.899	0.926

Table 1 is a list of uniquely detected, significantly increased, non-detected, and significantly decreased brown adipose tissue proteome at 4-month after ovariectomy, as ranked by *p*-values. ^2^ Sham and Ovx expression ratios were the Mean ± SD of peptides quantified in each sample expressed relative to the pooled internal standard. ^3^ *p*-values were analyzed by paired Student's *t*-test. Fold difference relative to Sham values was reported and *p-*values were determined from log-transformed data using Student's independent *t*-tests. ^4^ The effect size was calculated by pooled SD, and cohen's d. ^5^ The sample size of 8 rats in each group was validated by post hoc power analysis with α = 0.05 and power greater than 0.9.

## Abbreviations

AMPK: adenosine monophosphate-activated protein kinase
BAT: Brown adipose tissue
C/EBP-β: CCAAT enhancer binding protein beta
E2F1: E2F transcription factor 1
FDR: false discovery rate
FOXC2: forkhead box C2
FXR: farnesoid X receptor
IPA: Ingenuity Pathway Analysis
KLF11: Kruppel-like factor 11
LXR: liver X receptor
Ovx: ovariectomy
PFP: parametrial fat pad
PPARγ: peroxisome proliferator-activated receptor-γ
RFP: retroperitoneal fat pad
RXR: retinoid X receptor
SD: standard deviation
Sham: sham-operation
SREBF1: sterol regulatory element binding transcription factor 1
TCA: tricarboxylic acid
UCP1: uncoupling protein 1
